# Do Admitted COVID-19-Positive Patients on Anticoagulation Therapy Have a Reduced Hospital Stay and Disease Severity?

**DOI:** 10.7759/cureus.23382

**Published:** 2022-03-22

**Authors:** Haroon Nawaz, Ayesha Choudhry, William J Morse, Oskar Zarnowski, Harsh Patel, Harshad Amin

**Affiliations:** 1 Internal Medicine, Westside Regional Medical Center, Plantation, USA; 2 Research, Fatima Jinnah Medical University, Lahore, PAK; 3 Research, Ross University School of Medicine, Miramar, USA; 4 Research, Nova Southeastern University Dr. Kiran C. Patel College of Osteopathic Medicine, Fort Lauderdale, USA; 5 Hematology and Oncology, Westside Regional Medical Center, Plantation, USA

**Keywords:** packed red blood cell transfusion, duration of hospital stay, anti-coagulation, coagulation abnormalities, covid 19

## Abstract

Background

As of December 2021, the coronavirus disease 2019 (COVID-19) pandemic has resulted in the deaths of over 5 million people. It is known that infection with this virus causes a state of hypercoagulability. Because of this, there has been considerable debate on whether or not patients should be placed on anticoagulation prophylaxis/therapy. The goal of our project was to shed light on this topic by examining the effects of preexisting anticoagulation therapy in COVID-19 patients on disease severity (measured by blood clot readmissions, transfusion counts, and length of hospital stay). In this retrospective cohort study, we conducted an analysis based on data from 30,076 COVID-19-positive patients’ electronic medical records.

Materials and methods

This is a retrospective cohort study. Patients included in this study were identified from the HCA Healthcare corporate database. Registry data was sourced from HCA East Florida hospitals. All patients included in this study were COVID-19 positive via polymerase chain reaction (PCR) or rapid antigen testing on admission and over age 18. A total of 30,076 patients were included in this study with hospital admission dates from March 1, 2020 to June 30, 2021. The analysis examined the relationship between age, sex, blood clot history, and most importantly current anticoagulation status on COVID-19 disease severity (through blood clot readmissions, length of stay, and transfusion count). Blood clot readmissions were analyzed with a logistic regression model while the length of hospital stay and transfusion count were analyzed with a linear regression model.

Results

Our analysis revealed that the odds of experiencing a blood clot readmission is 2.017 times more likely in patients already on anticoagulation therapy compared to those who were not (p = 0.0024). We also found that patients on anticoagulation therapy had a hospital stay of 6.90 days longer on average than patients not on anticoagulation therapy (p < 0.0001). Finally, patients on anticoagulation therapy had, on average, 0.20 more blood transfusions than patients not on anticoagulation therapy (p < 0.001).

Conclusion

While these findings may be affected by the underlying conditions of those on preexisting anticoagulation therapy, they provide valuable insight into the debate on whether COVID-19-positive patients should be anticoagulated on admission to a hospital.

## Introduction

The coronavirus disease 2019 (COVID-19) pandemic caused by the severe acute respiratory syndrome coronavirus 2 (SARS-CoV-2) has affected over 250 million people as of December 2021. It has resulted in the deaths of over 5 million people [1]. COVID-19 often presents with flu-like symptoms, with the most common being fever and cough [2]. While many patients endure a mild disease course, around 14% progress to severe disease involving hypoxia, dyspnea, or >50% lung involvement on imaging. The fatality rate among the general population is around 2%, and about 15% in patients >80 years old [1,3]. While respiratory dysfunction remains the hallmark of COVID-19, there is mounting evidence that it also induces a hypercoagulable state proportional to disease severity [4-7]. Patients often present with abnormal coagulation lab values including D-dimer and activated partial thromboplastin time (aPTT) [8]. This hypercoagulability is likely caused by the direct action of the virus on vascular endothelium and platelets, among other tissues, and is potentiated by indirect immune activation [9,10]. Venous thromboembolism, for example, is estimated to affect 25% of COVID-19 patients in the ICU, often despite prophylactic anticoagulation therapy [11]. The question remains whether or not patients with COVID-19 should be treated with prophylactic/therapeutic doses of anticoagulants, and if this treatment improves clinical outcomes. This retrospective study aims to shed light on this topic by evaluating the effects of preexisting anticoagulant therapy on the duration of hospital stay and the severity of the disease.

## Materials and methods

This is a retrospective cohort study. Patients included in this study were identified from the HCA Healthcare corporate database. Registry data was sourced from HCA East Florida hospitals. A total of 30,076 patients were included in this study with hospital admission dates from March 1, 2020 to June 30, 2021. All patients included in this study were COVID-19 positive via polymerase chain reaction (PCR) or rapid antigen testing on admission and over age 18. Two cohorts of patients were created: an anticoagulation therapy cohort included 14,557 patients who were already taking any anticoagulation therapy for a preexisting condition; and a control cohort which included 15,519 not on anticoagulation therapy.

Data was collected by extraction from electronic medical records. Data collected included age, sex, race, mortality, previous blood clot history, readmission for new blood clot 90 days post-COVID-19 diagnosis, quantity of blood transfusions post-COVID-19 diagnosis, anticoagulation status, and length of hospital stay. 

Logistic regression and linear regression models examined the relationship between age, sex, blood clot history, and most importantly current anticoagulation status on COVID-19 disease severity (through blood clot readmissions, length of stay, and transfusion count). Logistic regression is a type of statistical analysis used to predict the odds of a desired association. Requirements for the proper construction of the model include: a binary, dependent variable compared alongside one or more continuous or categorical, predictor variables. Blood clot readmissions were analyzed with a logistic regression model, while the length of hospital stay and transfusion count were analyzed with a linear regression model. 

This study protocol was determined to be exempt or excluded from Institutional Review Board (IRB) oversight in accordance with current regulations and institutional policy at HCA Florida (Internal Reference Number 2021-311). It was determined that written consent was unnecessary due to de-identified personal health information being used. Both raw and analyzed data is available from the corresponding author at reasonable request. 

## Results

Baseline statistical information

A total of 30,076 COVID-19 PCR/rapid antigen positive patients admitted to HCA hospitals from March 1, 2020 to June 30, 2021 were included in our analysis. Of these, 48.40% were taking anticoagulation for a preexisting condition and 51.60% were not. Please see Table 1 for statistics information on the entire data set.

**Table 1:** Statistical information gathered from electronic medical records BC = Blood Clot; LOS = Length of Stay

	Cumulative (n=30076)
Mean Age	55.22 Years
Male Sex	47.97%
Race	
White	59.31%
Black	28.46%
Other	12.23%
Mortality	5.63%
BC History	1.86%
Mean Transfusion Count	0.17
Mean LOS	4.95 Days
BC Readmissions	0.35%
Anticoagulation Therapy	48.40%

Table 2 gives the logistic regression analysis for blood clot readmissions within 90 Days after COVID-19 diagnosis.

**Table 2:** Analysis of maximum likelihood estimates The regressors displaying a significant relationship with the outcome variable are in bold. ChiSq = Chi-Square; BC = Blood Clot; AC = Anticoagulation

Parameter	Estimate	Standard Error	Wald Chi-Square	Pr>ChiSq
Intercept	-7.4918	0.3913	366.6573	< .0001
Age (1 Year Older Difference)	0.0210	0.00581	13.0986	0.0003
Male Sex	0.2440	0.1973	1.5300	0.2161
BC History	0.4856	0.4659	1.0863	0.2973
AC Therapy	0.7018	0.2316	9.1854	0.0024

When determining the validity of the odds ratio point estimate, it is useful to look at the 95% Wald Confidence Limits in Table 3. If the range includes the value of 1.00, we can assume that there is no significant effect from that variable. 

**Table 3:** Odds ratio estimates Significant values are in bold. BC = Blood Clot; AC = Anticoagulant

Effect	Point Estimate	95% Wald Confidence Limits	
Age (1 Year Older Difference)	1.021	1.010	1.033
Male Sex	1.276	0.867	1.879
BC History	1.625	0.652	4.050
AC Therapy	2.017	1.281	3.176

Out of the analyzed variables (age, sex, blood clot history, and anticoagulant therapy), only age and anticoagulant therapy had significant effects on blood clot readmissions within 90 days after COVID-19 diagnosis. 

The odds of experiencing a blood clot readmission within 90 days after a COVID-19 diagnosis is more likely for older patients than younger patients, assuming all the other variables are held constant. For each increase of one year in age, a patient’s odds of experiencing a blood clot readmission is 1.02 times as likely to occur. One may be 95% confident that a patient’s odds to experience a blood clot readmission within 90 days after a COVID-19 diagnosis are between 1.01 and 1.03 times higher as opposed to those who are one year younger.

The odds of experiencing a blood clot readmission is 2.017 times as likely for patients on anticoagulation therapy as opposed to patients not on anticoagulation therapy, assuming all other variables are held constant. In short, one may be 95% confident that a patient's odds to experience a blood clot readmission within 90 days after a COVID-19 diagnosis are between 1.28 and 3.18 times higher if they are on anticoagulation therapy than if they are not on anticoagulation therapy. 

Linear regression analysis for length of hospital stay

Within Table 4, one is able to locate the categorical variables added to the model and the various levels found within each predictor. Note the coding of 0 and 1 and their respective delineations.

**Table 4:** Class level information M = Male; F = Female

Class	Levels	Values
Sex	2	M F
Blood Clot History	2	1-present 0-not present
Anticoagulation Therapy	2	1-present 0-not present

With a p-value of <.0001, the model for length of stay is statistically significant. Thus, we are able to further analyze and make interpretations off the model (Table 5).

**Table 5:** Length of stay model analysis Statistically significant values are shown in bold. DF = Degrees of Freedom

Source	DF	Sum of Squares	Mean Square	F Value	Pr > F
Model	4	537233.523	134308.381	2260.44	<0.0001
Error	30071	1786723.623	59.417		
Corrected Total	30075	2323957.146			

Table 6 shows the final results of our analysis.

**Table 6:** Final outcomes of linear regression for hospital length of stay Statistically significant p-values are shown in bold.

Parameter	Estimate	Standard Error	t-value	Pr > |t|
Intercept	-2.186841176	0.13872733	-15.76	< .0001
Age (1 Year Older Difference)	0.060874554	0.00246337	24.71	<.0001
Male Sex	0.724650724	0.08930280	8.11	<.0001
Female Sex	0.000000000	-	-	-
Blood Clot History (1-Present)	4.635008175	0.33247285	13.94	<.0001
Blood Clot History (0-Not Present)	0.000000000	-	-	-
Anticoagulation Therapy (1-Present)	6.901757786	0.09679239	71.30	<.0001
Anticoagulation Therapy (0-Not Present)	0.000000000	-	-	-

All of the analyzed variables (age, sex, blood clot history, and anticoagulant therapy) had significant effects on hospital length of stay after COVID-19 diagnosis. 

With a p-value of <0.0001 and a coefficient estimate of 0.72, one has reason to believe that length of hospital stay post-COVID-19 diagnosis is statistically significant in the positive direction for male patients. Female patients have a shorter length of stay and male patients have a longer length of stay. The average difference in length of stay between male and female patients is 0.72. So, compared to females, we would expect males to have a length of stay 0.72 days longer, on average, maintaining all other predictors remain constant.

With a p-value of <0.0001 and a coefficient estimate of 0.06, one has reason to believe that length of hospital stay post-COVID-19 diagnosis is statistically significant in the positive direction for older patients (calculated per 1 year older). Older patients have a longer length of stay, and younger patients have a shorter length of stay. The length of hospital stay post-COVID-19 diagnosis is longer by 0.06 days.

With a p-value of <0.0001 and a coefficient estimate of 4.64, one has reason to believe that length of hospital stay post-COVID diagnosis is statistically significant in the positive direction for those with a blood clot history. Those with a blood clot history have a longer length of stay, and those without a blood clot history have a shorter length of stay. The average difference in length of stay between those with a blood clot history and those without a blood clot history is 4.64. We would expect patients without a blood clot history to have a length of stay 4.64 days shorter than those with a blood clot history, on average, maintaining all other predictors remain constant.

With a p-value of <0.0001 and a coefficient estimate of 6.90, one has reason to believe that length of hospital stay post-COVID diagnosis is statistically significant in the positive direction for those on anticoagulation therapy. Those on anticoagulation therapy have a longer length of stay, and those not on anticoagulation therapy have a shorter length of stay. The average difference in length of stay between those on anticoagulation therapy and those not on anticoagulation therapy is 6.90. So, compared to those on anticoagulation therapy, we would expect patients not on anticoagulation therapy to have a length of stay 6.90 days shorter, on average, maintaining all other predictors remain constant.

Linear regression analysis for transfusion count post-covid admission

Within Table 7, one is able to locate the categorical variables added to the model and the various levels found within each predictor. Notice the coding of 0 and 1 and their respective delineations.

**Table 7:** Class level information M = Male; F = Female

Class	Levels	Values
Sex	2	M F
Blood Clot History	2	1-present 0-not present
Anticoagulation Therapy	2	1-present 0-not present

With a p-value of <.0001, the model for blood transfusions is statistically significant. Thus, we are able to further analyze and make interpretations off the model (Table 8).

**Table 8:** Transfusion count model analysis Statistically significant values are shown in bold. DF = Degrees of Freedom

Source	DF	Sum of Squares	Mean Square	F Value	Pr > F
Model	4	445.66023	111.41506	57.32	<0.0001
Error	30071	58449.74192	1.94372		
Corrected Total	30075	58895.40215			

Table 9 shows the final results of our analysis.

**Table 9:** Final outcomes of linear regression for transfusion count Statistically significant p-values are shown in bold.

Parameter	Estimate	Standard Error	t-value	Pr > |t|
Intercept	-.0348339058	0.02509138	-1.39	0.1651
Age (1 Year Older Difference)	0.0014007089	0.00044555	3.14	0.0017
Male Sex	0.0483650820	0.01615205	2.99	0.0028
Female Sex	0.000000000	-	-	-
Blood Clot History (1-Present)	0.1337559599	0.06013382	2.22	0.0261
Blood C lot History (0-Not Present)	0.000000000	-	-	-
Anticoagulation Therapy (1-Present)	0.2009988556	0.01750668	11.48	<.0001
Anticoagulation Therapy (0-Not Present)	0.000000000	-	-	-

With a p-value of <.0001 and a coefficient estimate of 0.05, one has reason to believe that transfusion count post-COVID-19 diagnosis is statistically significant in the positive direction for male patients. Female patients have less blood transfusions, and male patients have more blood transfusions. The average difference in blood transfusions between male and female patients is 0.05. So, compared to females, we would expect males to have 0.05 more blood transfusions, on average, maintaining all other predictors remain constant.

With a p-value of <.0001 and a coefficient estimate of 0.001, one has reason to believe that transfusion count post-COVID-19 diagnosis is statistically significant in the positive direction for older patients (calculated per 1 year older). Older patients have more blood transfusions, younger patients have less blood transfusions. For each additional year of age, the transfusion count post-COVID diagnosis increases by 0.001.

With a p-value of <.0001 and a coefficient estimate of 0.13, one has reason to believe that transfusion count post-COVID diagnosis is statistically significant in the positive direction for those with a blood clot history. Those with a blood clot history have more blood transfusions, and those without a blood clot history have fewer blood transfusions. The average difference in blood transfusion number between those with a blood clot history and those without a blood clot history is 0.13. So, compared to those without a blood clot history, we would expect patients with a blood clot history to have 0.13 more blood transfusions, on average, maintaining all other predictors remain constant. 

With a p-value of <.0001 and a coefficient estimate of 0.20, one has reason to believe that transfusion count post-COVID-19 diagnosis is statistically significant in the positive direction for those on anticoagulation therapy. Those on anticoagulation therapy have more blood transfusions, and those not on anticoagulation therapy have fewer blood transfusions. The average difference in blood transfusions between those on anticoagulation therapy and those not on anticoagulation therapy is 0.20. So, compared to those not on anticoagulation therapy, we would expect patients on anticoagulation therapy to have 0.20 more blood transfusions, on average, maintaining all other predictors remain constant.

## Discussion

Our study shows that COVID-19 patients on preexisting anticoagulation therapy are 2.017 times more likely to have blood clot readmission, have hospital stay 6.90 days longer, and, on average, require 0.20 more blood transfusions compared to those not on preexisting anticoagulation therapy. To this day, our knowledge and evidence of using routine anticoagulation therapy in COVID-19 patients to improve outcomes are limited [12].

Although the hallmark of COVID-19 remains to be respiratory dysfunction, COVID-19-positive patients are more likely to experience coagulopathy, increasing their risk for venous thromboembolism, deep venous thrombosis, pulmonary embolism, and disseminated intravascular coagulation. Multiple studies have evaluated COVID-19 patients taking anticoagulation therapy and have produced highly variable results. For example, a study by Fröhlich et al. found that COVID-19 hospitalized patients receiving direct oral anticoagulants (DOACs) had improved outcomes [13].^ ^Another study by Chocron et al. found that prior to hospitalization, anticoagulation therapy was linked to improved outcomes [14]. Conversely, the findings of other studies by Rivera-Caravaca et al. and Tremblay et al. found that there was no improved clinical outcome observed in patients on anticoagulation therapy at the time of admission, but rather increased mortality rates and decreased rate of survival [15,16].

One of our study’s limitations is the retrospective cohort study design, which could have affected the results due to potential confounding variables. For example, differing classes of anticoagulants, underlying comorbidities, and vaccination status were unaccounted for. An important strength of our study is that, to our knowledge, we have one of the largest cohorts of patients on preexisting anticoagulation therapy, therefore, increasing the reliability of our results. 

## Conclusions

There is still no definitive answer on whether hospitalized COVID-19 patients should be treated with therapeutic anticoagulation. However, our findings show there is a significant association between ongoing anticoagulation use in COVID-19 patients and disease severity. Our hope is that our findings from this research study can provide valuable insight into the debate on whether COVID-19-positive patients should be started on anticoagulation therapy upon admission to a hospital.

## References

[1] (2021). Weekly operational update on COVID-19 - 21 December 2021. https://www.who.int/publications/m/item/weekly-operational-update-on-covid-19---21-december-2021.

[2] Guan WJ, Ni ZY, Hu Y (2020). Clinical characteristics of coronavirus disease 2019 in China. N Engl J Med.

[3] Wu Z, McGoogan JM (2020). Characteristics of and important lessons from the coronavirus disease 2019 (COVID-19) outbreak in China: summary of a report of 72 314 cases from the Chinese Center for Disease Control and Prevention. JAMA.

[4] Terpos E, Ntanasis-Stathopoulos I, Elalamy I (2020). Hematological findings and complications of COVID-19. Am J Hematol.

[5] Benhamou D, Keita H, Ducloy-Bouthors AS (2020). Coagulation changes and thromboembolic risk in COVID-19 obstetric patients. Anaesth Crit Care Pain Med.

[6] Nascimento JH, Gomes BF, Carmo Júnior PR (2020). COVID-19 and hypercoagulable state: a new therapeutic perspective. Arq Bras Cardiol.

[7] Al-Ani F, Chehade S, Lazo-Langner A (2020). Thrombosis risk associated with COVID-19 infection. A scoping review. Thromb Res.

[8] Kollias A, Kyriakoulis KG, Dimakakos E, Poulakou G, Stergiou GS, Syrigos K (2020). Thromboembolic risk and anticoagulant therapy in COVID-19 patients: emerging evidence and call for action. Br J Haematol.

[9] Sardu C, Gambardella J, Morelli MB, Wang X, Marfella R, Santulli G (2020). Hypertension, thrombosis, kidney failure, and diabetes: is covid-19 an endothelial disease? A comprehensive evaluation of clinical and basic evidence. J Clin Med.

[10] Page EM, Ariëns RA (2021). Mechanisms of thrombosis and cardiovascular complications in COVID-19. Thromb Res.

[11] Tal S, Spectre G, Kornowski R, Perl L (2020). Venous thromboembolism complicated with COVID-19: what do we know so far?. Acta Haematol.

[12] Tang N, Li D, Wang X, Sun Z (2020). Abnormal coagulation parameters are associated with poor prognosis in patients with novel coronavirus pneumonia. J Thromb Haemost.

[13] Fröhlich GM, Jeschke E, Eichler U (2021). Impact of oral anticoagulation on clinical outcomes of COVID-19: a nationwide cohort study of hospitalized patients in Germany. Clin Res Cardiol.

[14] Chocron R, Galand V, Cellier J (2021). Anticoagulation before hospitalization is a potential protective factor for COVID-19: insight from a french multicenter cohort study. J Am Heart Assoc.

[15] Rivera-Caravaca JM, Núñez-Gil IJ, Vivas D (2021). Clinical profile and prognosis in patients on oral anticoagulation before admission for COVID-19. Eur J Clin Invest.

[16] Tremblay D, van Gerwen M, Alsen M (2020). Impact of anticoagulation prior to COVID-19 infection: a propensity score-matched cohort study. Blood.

